# Under the Microscope: A Case Report of Thoracic SMARCA4-Deficient Undifferentiated Tumor with Review of the Literature

**DOI:** 10.5146/tjpath.2023.12965

**Published:** 2024-05-18

**Authors:** Manasi Mundada, Khalid Abdul Mannan, Divya Vasu, Faiq Ahmed, Suseela K

**Affiliations:** Department of Pathology, Basavatarakam Indo American Cancer Hospital & Research Institute, Hyderabad, India

**Keywords:** SMARCA4-deficient undifferentiated tumor, Lung, Thorax, BRG1, Metastasis

## Abstract

**Objective:** SMARCA4-deficient undifferentiated tumor (SMARCA4-UT) is a highly malignant neoplasm with an undifferentiated or rhabdoid phenotype, posing a diagnostic challenge. This case report aims to create awareness about this rare neoplasm while dealing with cases presenting with undifferentiated morphology.

**Case Report:** A 55-year-old gentleman with constitutional symptoms and lymphadenopathy. Imaging revealed a mass lesion in the right upper lobe of the lung. A biopsy of the cervical lymph node showed diffusely effaced architecture replaced by sheets of undifferentiated pleomorphic cells with vesicular nuclei, prominent nucleoli, eosinophilic cytoplasm, and multiple necrotic foci. An extensive immunohistochemistry (IHC) panel was applied, which showed positivity for synaptophysin, vimentin, and focal CD34 and EMA expression. Other markers like pan-cytokeratin, p40, TTF1, CD56, INSM1, calretinin, CD45, SOX10, S100, CD30, CD117, SMA, and Desmin were negative, with INI1 retained. The IHC panel excluded the morphological differentials of carcinoma, lymphoma, rhabdomyosarcoma, melanoma, and germ cell tumor. Further literature review led to the possibility of the SMARCA4-UT entity, which had a morphology and IHC profile similar to the present case. Testing for SMARCA4 (BRG-1) by IHC showed a complete loss in the tumor cells, favoring the diagnosis of Thoracic SMARCA4-deficient undifferentiated tumor (SMARCA4-UT).

**Conclusion:** SMARCA4-UTs are rare, highly aggressive, and poorly differentiated thoracic tumors. Recognizing them is vital as there is potential for therapeutic interventions such as immunotherapy and SMARCA4-targeted therapies, offering promising prospects for the future.

## INTRODUCTION

SMARCA4-deficient thoracic undifferentiated tumors (SMARCA4-UT) represent a recently discovered and characterized pathological entity. This disorder was initially documented by Loarer et al. in 2015 (1). SMARCA4-UT is primarily distinguished by the inactivation of the SMARCA4 gene situated at 19p13, which encodes the Brahma related gene-1 (BRG1) protein. This protein is a critical constituent of the switch/sucrose-nonfermenting (SWI/SNF) chromatin remodeling complex. SMARCA4-UT is strongly associated with smoking and displays a molecular profile similar to that of smoking-related non-small cell lung carcinoma (NSCLC) and also exhibits a focal expression of NSCLC markers such as TTF1 and p40, as reported by Rekhtman et al. suggesting that SMARCA4 deficient thoracic sarcomas are conceptually similar to sarcomatoid carcinomas and have undergone epithelial-mesenchymal transition (2).

SMARCA4-UT predominantly affects young and middle-aged individuals, with a slight male predominance. These tumors exclusively occur in the thoracic region and commonly present as masses in the mediastinum, lung, and/or pleura. The clinical prognosis of this disease is known to be unfavorable (3). Morphologically, the tumor displays an undifferentiated and/or rhabdoid phenotype and exhibits the expression of one or more stem cell markers, including CD34, SOX2, and SALL4. Thymic, lung, and mesothelial markers are absent, and there is a complete loss of BRG1 protein, as confirmed by immunohistochemistry (IHC) (4,5).

Herein, we present a case report of SMARCA4-deficient thoracic undifferentiated tumor (SMARCA4-UT) that posed challenges in its diagnosis based on examination of the lymph node biopsy specimen. The encountered difficulties in utilizing a panel of immunohistochemistry (IHC) markers and conducting a comprehensive literature review to establish an accurate diagnosis for this particular case of SMARCA4-UT are presented.

## CASE REPORT

**Figure 1:**
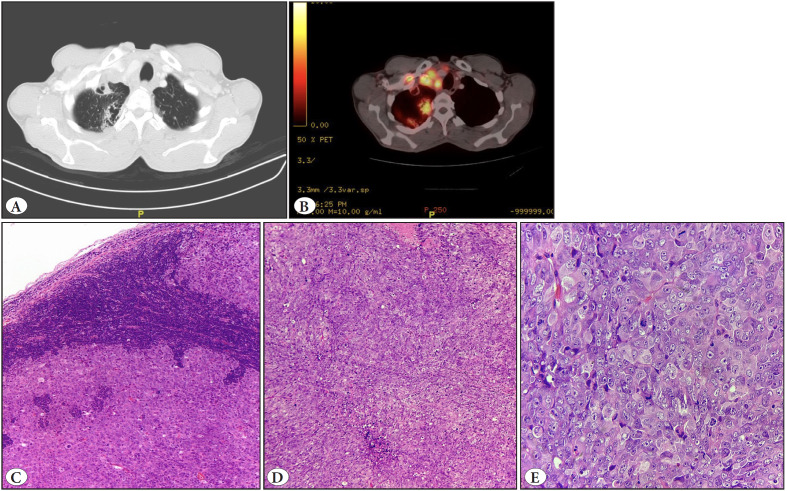
**A)** CT scan showing irregular thick-walled cavitary lesion measuring 22x21 mm with pleural tag in right lung apex. **B)** The PET-CT scan showed a hypermetabolic irregular thick-walled cavitary lesion in the right lung apex (SUV max 7.8) and multiple enlarged conglomerated lymph nodes. **C,D)** H&E stained sections of lymph node show sheets of undifferentiated to epithelioid cells (100x). **E)** High-power microphotograph showing tumor cells having vesicular nuclei, prominent nucleoli, and a moderate amount of eosinophilic cytoplasm with brisk mitosis (400x).

**Figure 2:**
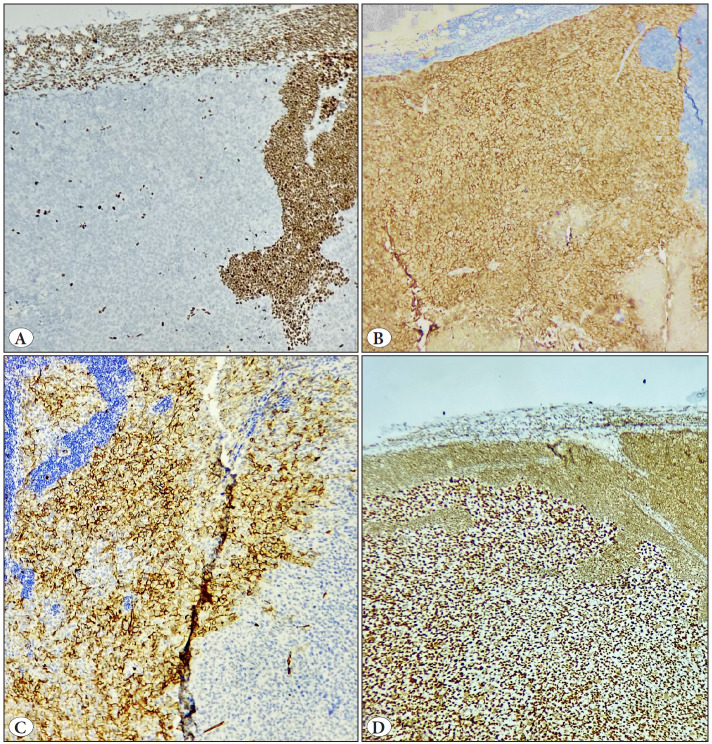
Immunophenotype features. **A)** BRG-1 immunostain shows a complete loss in tumor cells with lymphocytes as an internal control. **B)** Synaptophysin shows cytoplasmic positivity. **C)** CD34 expression is variable in tumor cells. **D)** INI-1 was retained.

A 55-year-old gentleman presented with symptoms of difficulty in breathing, a dry cough, intermittent fever, and enlarged lymph nodes on the right side of the neck. The computed tomography (CT) and positron emission tomography (PET) scan revealed the presence of a necrotic mass in the upper lobe of the right lung, measuring approximately 22×21 mm, with the surrounding lung tissue showing subpleural fibrosis and bronchiectatic changes (Figure 1). Other multiple enlarged and necrotic lymph nodes were detected in the mediastinum (measuring 64×49 mm), neck (measuring 26×24 mm), and the left paraaortic region. An excision biopsy was performed on a right cervical lymph node, revealing a complete loss of the normal nodal architecture replaced by neoplastic cells arranged in dyscohesive sheets, accompanied by extensive areas of necrosis. The neoplastic cells exhibited a morphology ranging from epithelioid to undifferentiated cells, characterized by round to oval nuclei, vesicular chromatin, prominent nucleoli, and a moderate amount of eosinophilic cytoplasm with high mitotic count (Figure 1). Undifferentiated morphology raised the possibility of multiple differential diagnoses of poorly differentiated carcinoma, rhabdomyosarcoma, neuroendocrine carcinoma, melanoma, and lymphoma. Immunohistochemical (IHC) panel, including pan-cytokeratin (PCK), TTF1, p40, CD56, INSM1, Desmin, S100, SOX10, LCA, CD117, and CD30 markers, were tested, but they all yielded negative results. IHC staining for vimentin and synaptophysin was positive, CD34 and EMA showed very focal positivity in the tumor cells, while SMARCB1 (INI-1) was retained (Figure 2).

The diagnosis was confirmed by SMARCA4 (BRG-1) IHC, which showed a complete loss of SMARCA4 protein in the neoplastic cells. The treatment protocol included chemotherapeutic agents, paclitaxel, and carboplatin. CT scan done after three months of treatment revealed a moderate reduction in the size of the lung lesion and a slight decrease in lymph node volume with an increase in necrotic tissue.

## DISCUSSION

Thoracic SMARCA4-UT tumors are rare neoplasms, with approximately 100 cases reported globally (1,3,4,6). They commonly manifest during the fourth and fifth decades of life, exhibiting a broad age range (27 to 90 years). Furthermore, these tumors show a notable male predilection, with a male-to-female ratio of 9:1, and are strongly associated with smoking (4). Most patients present with symptoms attributable to the mass effect and compression exerted by the tumor on neighbouring structures. These symptoms typically include pain, dyspnea, cough, haemoptysis, and superior vena cava syndrome (4). Given the aggressive nature of the neoplasm, initial presentation with metastasis to lymph nodes, skeletal bones, brain, or the abdominal cavity/pelvis is not an uncommon occurrence (7). Radiological imaging scans indicate that these masses predominantly occur in the upper and middle mediastinum, frequently involving adjacent structures such as the esophagus, bronchus, thymus, and major blood vessels. Moreover, contiguous involvement of the lung parenchyma is frequently observed (8). Table 1 summarizes the clinicopathological features and survival data of cases in the published literature.

**Table 1:** 

**Author**	**No of patients**	**Age** **(median)**	**Sex**	**Smoking**	**Tumor size**	**Histomorphology**	**Metastatic disease**	**Treatment**	**Overall survival (OS)/progression-free survival (PFS)**
Present case (2023)	1	55y	Male	Yes	2.2cm	Undifferentiated to epithelioid	Present to lymph nodes	Chemotherapy – carboplatin + paclitaxel	No events post 4 cycles of chemotherapy
Rachidi et al. (9) (2023)	1	42y	Male	Yes	N/A	Epithelioid morphology	Present to lymph nodes	Chemotherapy – pazopanib	OS: less than 1 month
Shinno et al. (10) (2022)	18	53y	Male (predominant)	Yes (all)	N/A	N/A	Present in 13 (72%) patients	Immunotherapy (first or second line) in 12 patients	PFS: 1.8 months
Kawachi et al. (11) (2020)	3	64y	Female – 2 Male – 1	Yes (all patients)	6.4 cm	Undifferentiated round to plasmacytoid	Present to lymph node, bone, and brain	Atezolizumab + Bevacizumab + Paclitaxel + carboplatin	PFS: 2-17 months
Stewart et al. (12) (2020)	1	59y	Male	Yes	5 cm	Undifferentiated to rhabdoid	Absent	Lobectomy followed by adjuvant chemotherapy	N/A
Perret et al. (4) (2019)	30	48y	Female – 3 Male – 27	87% smokers	1.2-24 cm	Undifferentiated epithelioid to rhabdoid	Present – 20 Absent – 6 N/A – 4	Chemotherapy – 18 Surgery – 2 Surgery + chemo – 5 N/A – 5	OS (median) – 6 months
Kunimasa et al. (7) (2019)	2	45y	Males – 2	Yes (both)	5-11 cm	Undifferentiated to rhabdoid	Present to lymph nodes and bone	Chemotherapy + immune checkpoint inhibitors	OS – 11 months
Rekhtman et al. (2) (2019)	22	58y	Males – 16 Females - 6	Yes (95% of the patients)	9.2 cm	Undifferentiated round cell to rhabdoid dyscohesive cells	Present (91% patients) to lymph node, bone, adrenal gland	N/A	OS – 5.2 months
Lijima et al. (13) (2020)	1	76y	Male	Yes	N/A	Undifferentiated to rhabdoid	Present	Nivolumab – third line	Dramatic regression of tumor, efficacy maintained for 22 months
Takada et al. (14) (2019)	1	69y	Female	N/A	N/A	Polyhedral tumor cells with enlarged nuclei	Present to peritoneum and skin	Pembrolizumab – first line	Sustained partial response after 8 cycles
Sauter et al. (3) (2017)	12	59y	Males – 9 Females – 3	Yes – 5 No – 1 N/A – 6	N/A	Solid sheets of dyscohesive rhabdoid cells	Present in 7 patients	Chemotherapy -	OS (median) – 8 months
Kuwamoto et al. (15) (2017)	1	30y	Female	Yes	7.8cm	Undifferentiated dyscohesive cells with focal rhabdoid morphology	Present	Chemotherapy	N/A
Yoshida et al. (16) (2017)	12	39y	Males – 11 Female – 1	Yes (70%)	-	Dyscohesive epithelioid to rhabdoid cells	Present in 10 patients (83%)	Chemotherapy – 6 Surgery – 2 N/A - 4	OS (median) – 7 months

As exemplified by the current case, the histomorphology of thoracic tumors, regardless of metastasis, exhibits a high-grade undifferentiated or epithelioid to rhabdoid cell phenotype, characterized by a relatively uniform dyscohesive arrangement in sheets, accompanied by brisk mitosis and necrosis. Regarding immunoprofiling, these tumors typically exhibit stem cell markers such as CD34, SALL4, and SOX2. There is usually either an absence or varying expression of PCK, EMA, and neuroendocrine markers, except for synaptophysin, which may show positivity. Additionally, focal expression of non-small cell lung cancer (NSCLC) markers like p63, p40, and TTF1 may be observed, while INI-1 expression remains intact. Immunonegativity is observed for calretinin, WT1, NUT, CD30, ALK, HMB-45, Desmin, and LCA. The characteristic feature of these tumors is the complete loss or significant underexpression of SMARCA4 (BRG-1), along with SMARCA2 (BRM) loss in more than 95% of the cases (17).

Since the most common morphological presentation is as undifferentiated malignancy or with rhabdoid features, it warrants to rule out other mimics like carcinoma, lymphoma (large cell phenotype), germ cell tumor, melanoma, epithelioid sarcoma, and large cell neuroendocrine tumors. Differentiating these tumors on small biopsy is very challenging due to undifferentiated morphology and IHC promiscuity. As mentioned earlier, markers like CD34, PCK/EMA, and synaptophysin can be focally present and may not be helpful in small biopsies. Immunohistochemistry is of help if the before-mentioned markers are positive in a summative pattern. BRG1 IHC is helpful but it is not available at all centers. However, this antibody is now becoming essential in the repertoire of IHC panels to diagnose these neoplasms.

Another point to remember is distinguishing SMARCA4-UT from SMARCA4-deficient non-small cell lung cancer (SMARCA4-NSCLC), as the latter is relatively more prevalent. These two entities can be differentiated based on distinct histomorphological characteristics. SMARCA4-NSCLC typically presents with clear-cut adenocarcinoma (AdCC) features or, less frequently, squamous cell carcinoma (SCC). Additionally, the expression of CK7 and the absence or focal expression of CD34, SOX2, and SALL4 are helpful markers for differentiation (17,18). The loss of SMARCA4 (BRG-1) can exceptionally occur in tumors such as SMARCB1/INI1-retained epithelioid sarcoma (ES); in this context, the absence of SOX2 and SALL4 expression aids in distinguishing ES from SMARCA4-UT (19).

More prevalent entities such as large cell neuroendocrine carcinoma and small cell carcinoma may initially elicit misdiagnosis due to crush artifacts and tissue necrosis, along with the expression of synaptophysin and a high ki-67 index (2). These cases would derive an advantage from a composite approach involving a first-generation neuroendocrine marker (Synaptophysin and chromogranin) in conjunction with a second-generation neuroendocrine marker such as INSM1 and BRG-1 IHC loss to differentiate them from SMARCA4-UT.

Additional diagnostic techniques include next generation sequencing (NGS) in identifying SMARCA4 mutations. However, in some cases where the loss of BRG-1 expression was observed using IHC, it was not detected by NGS. This could be because of structural variations (translocations) involving the intronic regions. Fluorescence in-situ hybridization (FISH) also has a limited role in diagnosis due to the truncating mutations coupled with the loss of heterozygosity (LOH), which is frequently copy-neutral (accompanied by the duplication of the mutant allele) (2). Therefore, it is crucial to use IHC to diagnose SMARCA4 mutations accurately.

SMARCA4-UT are aggressive and are associated with a poor prognosis, with median overall survival ranging from 4 to 7 months (3,16). Improved outcomes have been reported in some instances of SMARCA4-UT treated with immunotherapy agents such as pembrolizumab (14), atezolizumab (11), and nivolumab (13). Notably, one documented case demonstrated a remarkable survival period of up to 22 months (20).

## CONCLUSION

SMARCA4-UT represents a rare subset of highly aggressive, poorly differentiated thoracic tumors primarily observed in middle-aged individuals with a history of smoking. These tumors are presently classified as epithelial tumors in the WHO classification of thoracic tumors (5). The diagnosis is established by integrating clinical and pathological features, with particular emphasis on undifferentiated morphology and loss of the BRG1 protein. Despite their unfavorable prognosis, there is potential for therapeutic interventions such as immunotherapy and SMARCA4-targeted therapies, offering promising prospects for the future.

## Conflict of Interest

The authors declare that they have no conflict of interest for this article.
